# Impact of Global Health Electives on US Medical Residents: A Systematic Review

**DOI:** 10.29024/aogh.2379

**Published:** 2018-11-05

**Authors:** Paul M. Lu, Elizabeth E. Park, Tracy L. Rabin, Jeremy I. Schwartz, Lee S. Shearer, Eugenia L. Siegler, Robert N. Peck

**Affiliations:** 1 Department of Internal Medicine, Perelman School of Medicine at the University of Pennsylvania, Philadelphia, PA, US; 2 Department of Medicine, Weill Cornell Medical College, New York, NY, US; 3 Department of Internal Medicine, Yale School of Medicine, New Haven, CT, US

## Abstract

**Background:**

The prevalence of global health in graduate medical education in the United States (US) has soared over the past two decades. The majority of US internal medicine and pediatric residency programs now offer global health electives abroad. Despite the prevalence of global health electives among US graduate medical programs today, challenges exist that may impact the experience for visiting trainees and/or host institutions. Previous reviews have predominately focused on experiences of undergraduate medical students and have primarily described positive outcomes.

**Objectives:**

The aim of this study was to summarize the overall impact of global health electives on US internal medicine, medicine-pediatric, and pediatric residents, paying specific attention to any negative themes reported in the literature.

**Methods:**

An Ovid MEDLINE and Ovid EMBASE literature search was conducted to identify studies that evaluated the effects of global health electives on US internal medicine, medicine-pediatric, and pediatric residents.

**Findings:**

Ten studies were included. Four positive themes emerged: (1) improvement of medical knowledge, physical examination, and procedural skills, (2) improvement in resourcefulness and cost-effectiveness, (3) improvement in cultural and interpersonal competence, and (4) professional and career development. Two negative themes were identified: (1) health risks and (2) safety risks.

**Conclusions:**

Global health electives provide a number of perceived benefits for US medical trainees; however, we importantly highlight health and safety concerns described while abroad. Global health educators should recognize the host of unique challenges experienced during a global health elective and investigate how to best mitigate these concerns. Incorporation of mandatory pre-, intra-, and post-elective training programs and establishment of universally adopted global health best practice guidelines may serve to address some the challenges visiting trainees encounter while abroad.

## Introduction

Global health is the study, research, and practice of improving health and achieving health equity for all people worldwide [1]. The inclusion of global health in graduate medical education in the United States (US) has soared over the past two decades [2,3]. For example, the percentage of pediatric residency programs in North America offering a global health elective more than doubled from 25% in 1995 [4] to 58% in 2013 [2]. A study of US internal medicine residency programs found that 57% offered a global health elective in 2009 [5].

Despite the prevalence of global health electives among US graduate medical programs today, challenges exist that may impact the experience for visiting trainees and/or host institutions. Graduate medical trainees from high-income countries (HICs), referred to in this paper as ‘visiting trainees’, have described both benefits and challenges of participating in global health electives, which often occur in low- and middle-income countries (LMICs). Literature pertaining to undergraduate medical students has demonstrated multiple concerns of global health electives including practicing medicine or performing procedures above the students’ level of competence [6,7,8], risks for occupational exposure [9], navigating different sociocultural norms [6,7,8,10], and power imbalances [6,10]. Furthermore, recent studies examining the perspective of LMIC host institutions have described multiple concerns of working with visiting trainees [11,12,13,14,15,16,17]. Specific concerns included lack of cultural sensitivity [12,13,14,15,17], inexperience with practicing medicine in a low-resource setting [17], resource drain [11,13], and preventable harms in patient care [11]. These concerns highlight the need for proactive effort by US medical institutions to ensure that their visiting trainees arrive adequately prepared prior to their global health elective and remain a net benefit to LMIC host institutions. Crump et al. summarized this issue well when he stated, “Global health training that benefits the trainee at the cost of the host is clearly unacceptable; mutual and reciprocal benefit, geared to achieving the program goals of all parties and aiming for equity, should be the goal [18].”

Thoughtful selection of participants and the use of pre-departure training may help prepare visiting trainees for the host of unique challenges experienced during a global health elective and mitigate the concerns described by LMIC host institutions. Best practice guidelines in global health and ethics have been published that provide concrete recommendations for visiting trainees to follow during abroad electives [18,19], but the lack of formal pre-departure training at many US medical institutions prevents these guidelines from becoming a part of visiting trainees’ curricula [2].

US medical institutions face pedagogical and ethical challenges in ensuring that their global health electives result in a safe and educational experience for their visiting trainees while remaining a net benefit to LMIC host institutions. Previous reviews have predominately focused on undergraduate medical students and primarily described benefits of global health electives with minimal attention paid to negative outcomes [20,21,22,23,24]. However, little remains known about the potential impacts of global health electives for visiting postgraduate trainees. The present systematic review specifically evaluates both the benefits and challenges of global health electives on visiting trainees, paying specific attention to any negative themes reported in the literature.

## Materials and Methods

We conducted a systematic review to summarize the benefits and challenges of global health electives for US-based internal medicine, medicine-pediatric, and pediatric residents. We followed the Preferred Reporting Items for Systematic Reviews and Meta-Analysis (PRISMA) statement guidelines during the preparation of our review [25].

### Search strategy

A literature search was conducted in June 2016 using Ovid MEDLINE (1946 to present) and Ovid EMBASE (1974 to present). Both in-process and other non-indexed citations were included. The search was not limited to a specific language, date, or publication status. Keywords used for the search included all combinations of (global and health, global and elective, international and health, or international and elective) AND (internal medicine or pediatric) AND (residen*). A cited reference search was conducted among included articles.

### Eligibility criteria

Studies were included if they met the following criteria: (1) global health elective occurred outside the US, (2) study participants were enrolled in or had graduated from an internal medicine, medicine-pediatric, or pediatric residency program in the US, and (3) study reported on the effect(s) of a global health elective on residents.

Exclusion was based on the following criteria: (1) unrelated to global health medical education, (2) subjects not graduate medical residents, (3) subjects non-US-based graduate medical residents, (4) subjects US-based graduate medical residents in another specialty, (5) effect(s) of global health elective on US graduate medical residents not reported, and (6) global health education occurred exclusively in the US.

### Study selection

Two authors (PL, EP) independently screened all titles and abstracts identified in the literature search. Full-text copies of all candidate articles were reviewed by both authors with disagreements resolved by discussion and consensus. When consensus could not be reached by the two reviewers, studies were sent to a third reviewer (TR) for final decision.

### Data extraction and analysis

Data was extracted using a template to record the name of the medical institution, description of global health program, learning objectives, description of pre-departure training, description of post-elective debriefing, host countries visited, study and comparison groups, assessment tool used, and summary of major findings. A thematic analysis was conducted to identify patterns in the data and themes for analysis. Using an inductive approach, we identified positive and negative effects of global health electives from each study. This involved multiple stages of data exploration and coding of all findings within the results and discussion sections of each study. The initial list of codes was then collated into broad themes. Four positive themes and two negative themes were identified, which were then labeled as ‘benefits’ and ‘concerns’. There were no a priori criteria for the types of effects measured.

## Results

### Search results

**Figure 1:**
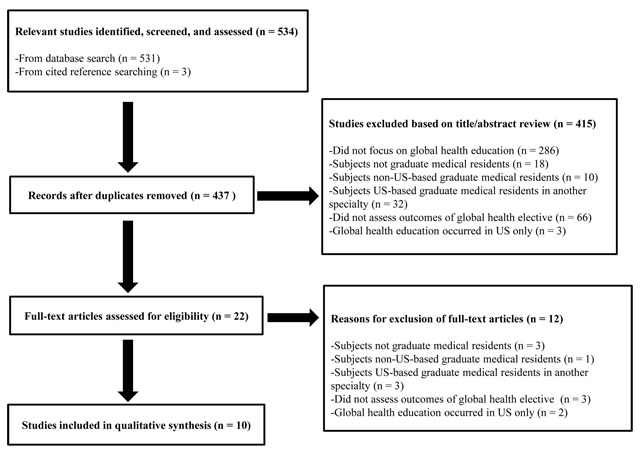
Selection Process Used in the Systematic Review.

Details of the study selection process are depicted in Figure 1. The original search returned 531 articles. Removal of duplicates and application of screening criteria resulted in 19 articles for full-text review. Three additional articles were identified through cited reference searching. Twenty-two articles were reviewed in full-text and 12 were excluded, leaving 10 articles for qualitative synthesis. Details of the 10 articles are listed in Table 1.

**Table 1:** Details of the Studies Included in the Systematic Review.

First author	Global health program	Learning objectives, pre-departure training, post-elective debriefing	Host countries	Study and comparison groups	Assessment tool (response rate)	Major findings

Miller et al [34]	Duke International Health Program Established: 1987 PGY: 2 or 3 Duration (weeks): 12 Funding: airfare covered	Learning objectives: learn global health and tropical medicine, provide cross-cultural experience, appreciate health care in another country Pre-departure training: not reported Post-elective debriefing: not reported	Brazil, China, Pakistan, Taiwan, Tanzania	Study group: internal medicine and med-peds residents (total n = 57) Comparison group: internal medicine and med-peds residents (total n = 123)	Survey using 7-point Likert scale with open ended question (91%)	Participants reported improvement of medical knowledge and tropical medicine and clinical diagnostic skills Minority of participants reported reduction in lab use Participants more likely to change career plans to academic medicine, public health, and include work abroad in the future Health consequences included traveler’s diarrhea, conversion of PPD, and malaria
Gupta et al [33]	Yale International Health Program Established: 1981 PGY: 2 or 3 Duration (weeks): 4–8 Funding: airfare, housing, and partial living expenses covered	Learning objectives: observe primary care in diverse cultural settings, promote cost-consciousness with practice of the physical examination, engender sense of social responsibility Pre-departure training: not reported Post-elective debriefing: not reported	Fiji, Haiti, Tanzania, Zimbabwe	Study group: internal medicine residents (n = 96) Comparison group: internal medicine residents (n = 96)	Survey using 7-point Likert scale with open ended question (61%)	Participants reported improvement of the physical examination and believed it was under-used Participants more likely to plan to volunteer or work abroad in the future Participants more likely to work with patients on public assistance, immigrants, substance abusers, and HIV infected Participants more likely to work in public health and less likely in private practice; however, no difference in careers in general or subspecialty medicine
Nuckton et al [30]	Tulane Program in Community Medicine Established: not reported PGY: 3 or 4 Duration (weeks): 4 Funding: airfare and living expenses partially covered	Learning objectives: observe healthcare in another country, learn new pathology, develop cultural competency Pre-departure training: reviewed history and endemic conditions to host country, counseled on health precautions Post-elective debriefing: not reported	Guatemala, Belize	Study group: med-peds residents (n = 3) Comparison group: none	Log of clinical cases (100%)	Total of 803 cases logged, most common disease category was gastrointestinal, accounting for 26% of cases Intestinal parasites represented 11% of cases and included: ascariasis, chilomastix, entamoeba, endolimax, giardia, hymenolepis, plasmodium, taenia, and trichomonas Health consequences included diarrhea with two participants treated for intestinal parasites (one confirmed, one presumed)
Federico et al [28]	University of Colorado Department of Pediatrics Global Health Elective Established: 2000 PGY: 2 or 3 Duration (weeks): 4 Funding: housing and in-country transportation covered	Learning objectives: observe health care and common medical and public health problems in another country, learn medicine and social interactions in a second language Pre-departure training: monthly discussions on history, culture, and language of host country, tropical medicine, safety precautions Post-elective debriefing: not reported	Guatemala, Peru	Study group: pediatric residents (n = 13) Comparison group: none	Log of clinical cases (93%)	Total of 890 cases logged with 18% of cases related to a disease never seen and 6% of cases to a disease in an advanced stage never before seen Thirty-two percent of cases related to infectious diseases and included: amebiasis, ascariasis, brucellosis, cysticercosis, echinococcus, leishmaniasis, leptospirosis, strongyloidiasis, tetanus, toxocariasis, and typhoid
Castillo et al [27]	Cincinnati Children’s Hospital Medical Center Global Health Scholars Program Established: 2007 PGY: 2–4 Duration (weeks): 2–4 Funding: not reported	Learning objectives: not reported Pre-departure training: counseled on cross-cultural communication, safety, legal requirements Post-elective debriefing: conducted but not described	Cambodia, Dominican Republic, Haiti, Honduras, Japan, Kenya, South Africa, Swaziland, Tanzania	Study group: pediatric and med-peds residents (total n = 13) Comparison group: none	Reflective journal passages coded for qualitative analysis using the ASTMH three competency domains^a^ (100%)	Participants reflected on 2 of 3 ASTMH competency domains: immigrant health (100%), burden of global disease (77%), and traveler’s medicine (0%) Two additional themes noted: humanitarianism (46%) and parental sacrifice (46%) Participants described improvement of cultural awareness and reflected on the need to build deeper relationships with patients, be better listeners, and be more patient Participants reported an increased desire to volunteer and advocate for future humanitarian outreach
Hau et al [29]	Weill Cornell Medical College Global Health Elective Established: 2006 PGY: 3 Duration (weeks): 4–6 Funding: airfare, vaccines, and visa covered	Learning objectives: not reported Pre-departure training: conducted but not described Post-elective debriefing: conducted in the host country but not described	Tanzania	Study group: internal medicine (n = 21) and pediatric (n = 18) residents (total n = 39) Comparison group: internal medicine (n = 14) and pediatric (n = 13) residents (total n = 27)	Survey using 7-point Likert scale with open ended question (58%)	Participants reported improved knowledge of global health, tropical medicine, and physical examination Participants rated routine laboratory testing as overused Majority of participants reported reduction in laboratory and/or radiologic tests No difference in career plans in general or subspecialty medicine
Gladding et al [35]	University of Minnesota Department of Pediatrics Global Health Track Established: 2005 PGY: 2 or 3 Duration (weeks): not reported Funding: not reported	Learning objectives: not reported Pre-departure training: not reported Post-elective debriefing: not reported	Bolivia, Cambodia, Ethiopia, Lebanon, Nicaragua, Panama, Tanzania, Uganda	Study group: pediatric (n = 21) and med-peds (n = 11) residents (total n = 32) Comparison group: none	Reflective essays analyzed for themes and grouped into 6 ACGME competencies^b^ (78%)	Greater than 90% of participants reflected on the ACGME competencies of: patient care, medical knowledge, and systems-based practice Greater than 50% of participants reflected on: practice-based learning and improvement, professionalism, interpersonal and communication skills, and profession and personal development (extra domain) Participants described improvement in communication with patients and health care workers despite language barriers as well as in working with interpreters Participants described use of local equipment that differed compared to those used in the US
Shull et al [31]	UCLA Department of Medicine Global Health Elective Established: 2008 PGY: 3 or 4 Duration (weeks): 3 Funding: airfare, housing, and in-country transportation covered	Learning objectives: provide knowledge and skills needed to treat patients in a developing country Pre-departure training: reviewed history, culture, and conditions endemic to the host country, learned local antiretroviral therapy protocols, counseled on health precautions Post-elective debriefing: conducted but not described	Malawi	Study group: internal medicine (n = 24) and med-peds (n = 9) residents (total n = 33) Comparison group: internal medicine and med-peds residents (n=not reported)	Survey using 4-point Likert scale with open ended question (83%)	Participants reported improved knowledge of HIV, tropical medicine, and physical examination One-third of participants reported reduced reliance on imaging Participants reported a broadened perspective on health care delivery in resource poor settings and increased awareness of cost-effectiveness Participants more likely to work in general internal medicine than nonparticipants Health consequences reported but not described
Arora et al [26]	UCLA Department of Pediatrics/UCLA School of Medicine Global Health Education Program Established: 2008 PGY: 3 Duration: not reported Funding: not reported	Learning objectives: not reported Pre-departure training: conducted but not described Post-elective debriefing: conducted but not described	Not reported	Study group: pediatric residents (n = 16) Comparison group: none	Debriefing interview with open-ended questions (84%)	Participants at partner sites (i.e., sites with established relationships) more likely to recommend abroad elective than those at nonpartner sites Participants at nonpartner sites experienced increased challenges like: limited educational opportunities, inaccurate resident expectations, and gaps in supervision One participant described their first attempt at a bone marrow biopsy without supervision from a fellow or attending physician
Balmer et al [32]	Baylor College of Medicine/Texas Children’s Hospital Global Child Health Program Established: 2010 PGY: 2–4 Duration (weeks): 4–52 Funding: not reported	Learning objectives: not reported Pre-departure training: not reported Post-elective debriefing: conducted but not described	Botswana, Lesotho, Malawi, Swaziland	Study group: categorical pediatric residents (n = 9) who spent 1 month abroad and global health pediatric residents (n = 9) who spent 12 months abroad (total n = 18) Comparison group: none	Semistructured interview with focus on reentry transitions using closed card sorting to assess emotional responses (53%)	Participants reported emotions of appreciation and inspiration but also expressed frustration and sadness Global health residents abroad for 12 months were more likely to report emotions of disconnection and confusion than categorical residents abroad for 1 month Categorical residents abroad for 1 month reported more invigoration compared to global health residents abroad for 12 months

Abbreviations: PGY, postgraduate year; Med-peds, medicine-pediatrics; PPD, purified protein derivative; HIV, Human Immunodeficiency Virus; ASTMH, American Society for Tropical Medicine and Hygiene; ACGME, Accreditation Council for Graduate Medical Education. ^a^ ASTMH 3 competency domains: (1) burden of global disease, (2) immigrant health, and (3) traveler’s medicine. ^b^ ACGME 6 competencies: (1) patient care, (2) medical knowledge, (3) practice-based learning and improvement, (4) interpersonal and communication skills, (5) professionalism, and (6) systems-based practice.

### Study characteristics and population

All studies were observational, cross-sectional studies. Nine academic institutions were represented in ten studies and included: four from the Midwest, two from the Northeast, two from the South, and one from the West. Learning objectives and pre-departure training varied widely across institutions. Six studies reported inclusion of pre-departure training [26,27,28,29,30,31] and five studies reported post-elective debriefing [26,27,29,31,32]. Nearly all global health electives took place in Africa, Asia, or Latin America and ranged from two to 52 weeks in duration. Funding for global health electives was reported in six studies and were partially paid for by the US academic institution or philanthropic support [28,29,30,31,33,34].

A total of 566 residents were analyzed for this review: 320 (57%) participants and 246 (43%) nonparticipants. Among participants, seven studies reported gender status with 156 (54%) females [27,29,31,32,33,34,35]. Three studies reported relationship status and found 113 (59%) were single [29,33,34]. Non-participants were residents from the same residency programs who did not participate in a global health elective. Three studies provided gender and relationship status for non-participants and found 91 (37%) were female and 76 (31%) single [29,33,34].

### Summary of benefits

All studies described a variety of self-reported positive effects related to participation in a global health elective. Four major themes were identified and included, (1) improvement of medical knowledge, physical examination, and procedural skills, (2) improvement in resourcefulness and cost-effectiveness, (3) improvement in cultural and interpersonal competence, and (4) professional and career development.

#### Improvement of medical knowledge, physical examination, and procedural skills

Eight studies reported on the effect of a global health elective on medical knowledge [27,28,29,30,31,33,34,35]. Participants reported a perceived positive effect on their knowledge of global health, human immunodeficiency virus (HIV), and/or tropical medicine. For example, a study examining case logs of pediatric residents in Latin America found that 158 of 890 (18%) cases pertained to a diagnosis the resident had never seen previously and that 50 of 890 (6%) cases had a familiar diagnosis but the stage of disease was more advanced than the resident had ever encountered previously [28]. Nearly one third of cases were categorized as infectious disease and included diseases rarely seen in the US, such as amebiasis, ascariasis, brucellosis, cysticercosis, echinococcus, leishmaniasis, leptospirosis, strongyloidiasis, tetanus, toxocariasis, and typhoid [28]. A study by Nuckton et al. evaluated case logs of residents traveling to Central America and found that gastrointestinal illness was the most common diagnosis, including 90 of 803 (11%) cases related to intestinal parasites [30]. Furthermore, two studies noted that participants learned to broaden their differential to better reflect the conditions endemic to the local region [31,35].

Five studies noted that residents self-reported improvement of their physical examination and/or procedural skills [29,31,33,34,35]. A study by Gupta et al. described residents’ attitudes toward the physical examination and noted that 77 (80%) participants of an elective abroad, compared with 66 (69%) nonparticipants, believed that physicians in the US under-utilized the physical examination (p = 0.03) [33].

#### Improvement of resourcefulness and cost-effectiveness

Six studies reported on the effect of an abroad elective on residents’ resourcefulness and awareness of cost-effectiveness [27,29,30,31,34,35]. Participants reported an increased sense of resourcefulness [27,29,35], a heightened awareness of cost-effectiveness [27,30,31], and a self-reported reduction in health care utilization on return to the US [29,31,34]. One study found that 39 (100%) participants versus 23 (85%) nonparticipants rated routine lab testing as overused (p = 0.01) [29].

#### Improvement of cultural and interpersonal competence

Four studies reported on the effect of a global health elective on cultural and interpersonal competence [27,30,31,35]. Gladding et al. noted that residents felt they learned to improve communication with patients and health care workers despite language and cultural barriers during their abroad elective [35]. Furthermore, participants reported improved skills in working with an interpreter. A study by Castillo et al. found that participants of abroad electives reported increased cultural awareness and reflected on the importance of building deeper relationships with patients, the need to be better listeners, and to be more patient with others [27].

#### Professional and career development

Six studies reported on the effect of a global health elective on professional and career development [27,29,31,33,34,35]. Four studies reported on careers or career plans and found participants were more likely to change career plans during residency towards general medicine [33,34], work in public health [33,34], and work with underserved patients [33]. A study by Shull et al. found that participants were more likely to work in general internal medicine than non-participants, 54% versus 24% (p < 0.01) [31]. Two studies found no difference in the careers or career plans in general internal medicine or subspecialty medicine when comparing participants and nonparticipants [29,33].

Participants also reported an impact on their professional development [27,35]. Castillo et al. reported that the majority of residents described their abroad experience as influential on their professional and personal development and reflected on a future desire to volunteer and advocate for humanitarian programs [27]. Another study found that participants of abroad electives solidified their interest in global health and gained personal insight and life perspective [35].

### Summary of concerns

Overall, fewer findings were reported that pertained to negative effects of global health electives. Six studies reported on a negative effect of a global health elective with two major themes noted, (1) health risks and (2) safety risks.

#### Health risks

Four studies reported on the effect of an abroad elective on residents’ health [30,31,32,34]. Three studies reported on the physical health [30,31,34] and one study on the mental health [32] consequences of a global health elective. In 1995, Miller et al. reported that 28 (49%) residents developed traveler’s diarrhea, eight (14%) required medical attention during or after their elective, three (5%) converted their purified protein derivative (PPD) status, and two (4%) were diagnosed with malaria [34]. In 1999, Nuckton et al. found that three (100%) residents developed diarrhea while abroad with two (67%) of the residents treated for intestinal parasites (one confirmed, one presumed) [30]. A more recent study from 2014 found that three (11%) residents reported physical illness during their abroad elective, though the type of illness or severity was not specified [31].

Balmer et al. examined the effect of a global health elective on mental health [32]. This study described the emotional responses of participants during interviews on re-entry to the US (i.e., reverse culture shock) and noted that 16 (89%) residents reported emotions of appreciation and 11 (61%) inspiration; however, nine (50%) residents reported sadness and seven (39%) frustration. Emotional responses were further characterized by residents in a dedicated global health residency program versus categorical residency program and by duration of travel. Global health residents abroad for 12 months were more likely to report emotions of disconnection (56% versus 11%) and confusion (44% versus 11%) whereas categorical residents abroad for one month reported increased invigoration (67% versus 11%) on return home.

#### Safety risks

Two studies reported on potential safety risks during global health electives [26,35]. Gladding et al. described use of local equipment that differed compared to those typically used in the US, particularly while performing procedures [35]. Arora et al. described an incident when a resident attempted and failed at their first bone marrow biopsy without supervision from a fellow or attending physician [26]. The study noted the incident occurred at a site without an established partnership with the sending US institution and demonstrated the potential negative effects on patient care and resident safety when trainees organize independent electives at nonpartner sites.

## Discussion

Resident participants of global health electives reported numerous perceived benefits from their abroad experiences, which we categorized into four themes. First, participants reported an improvement of medical knowledge. Second, participants reported improved physical examination and/or procedural skills, which likely contributed to the perceived improvement in cost-effectiveness and reduction in resource utilization on return home. Work in a developing country, often with limited resources, provides a unique opportunity for visiting trainees to learn and hone physical examination skills that often are neglected in the US and help develop skills of resourcefulness and cost-effectiveness [36,37]. Third, participants reported augmented cultural and interpersonal competence. Increasing globalization of the US population emphasizes the need for physicians who are culturally competent and effective communicators [20,38,39]. Abroad electives are one way to prepare trainees for the diverse patients they will encounter throughout their careers. Fourth, global health electives influence career choice and promote professional development. Consistent with prior reports, we found that participants of global health electives exhibited a future desire to volunteer, advocate for underserved patients, and work abroad [21,23,24,40,41,42].

However, it is important to consider the challenges experienced during global health electives. We describe health and safety concerns as reported by US internal medicine and pediatric residents. Emotional and mental health consequences are of particular concern in LMICs when visiting trainees face patient deaths and clinical complications that are often due to limited resources or other system challenges. Furthermore, ethical and safety risks to visiting trainees pose a significant concern. Lack of familiarity with local equipment may predispose to increased risk for occupational exposures. A study by Merlin et al. found that 25% of US medical students participating in a global health elective in sub-Saharan Africa experienced an occupational exposure during their trip [9]. This prompted incorporation of mandatory pre-departure simulation training, which significantly reduced the incidence of occupational exposures [43]. Other safety concerns among undergraduate medical students have also been reported in the literature including violence, motor vehicle accidents, sexual assault, and political unrest [44,45].

Despite the serious concerns raised in this review, we discovered that relatively few participants reported on the negative impact of abroad electives. Furthermore, the concerns we describe were reported from the perspective of US medical residents without input from host institutions. Future studies should further explore these concerns while investigating how to best mitigate the host of challenges visiting trainees encounter during a global health elective.

Pre-departure training provides an ideal opportunity to educate visiting trainees on the various challenges inherent to a global health elective as well as set goals and expectations prior to departure [43,46,47,48,49,50]. However, inclusion of pre-departure training has been far from universal. A recent survey of US pediatric residency programs found that only 66% of programs reported offering pre-departure training [2]. Prior guidelines have called for incorporation of pre-departure training programs and have provided frameworks for medical institutions to develop training curriculum [18,46,51]. Our findings align with current guidelines that recommend health and safety preparation as a component of pre-departure training. Guidelines from the Global Emergency Medicine Academy of the Society for Academic Emergency Medicine may serve as a helpful resource with specific recommendations on how to promote the health and safety of visiting trainees during an abroad elective [46]. In addition, incorporation of debriefing both during (i.e., intra-elective) and upon completion (i.e., post-elective) of global health electives may provide further benefits for visiting trainees. Intra-elective and post-elective debriefing provide opportunities for residents to reflect on the mixed emotions reported on return to the US, reinforce positive experiences, and allow for further personal and professional development [49]. Looking forward, we would propose that all global health electives be coupled with pre-, intra-, and post-elective training programs.

Furthermore, additional research and consensus meetings are needed to assess and validate which of the currently existing global health guidelines most effectively augment trainees’ education. In 2009, Drain et al. reported that no standardized global health curriculum existed and that the available curriculum varied widely throughout US medical centers [3]. We found this to be consistent with the global health programs described in our review which reported differing learning objectives and pre-departure training by program. However, over the past decade several groups have published guidelines for global health core competencies and ethics training [18,19,51,52,53,54]. Major groups include: the Association of Faculties of Medicine of Canada (AFMC) Resource Group on Global Health, Association of Schools of Public Health (ASPH), Consortium of Universities for Global Health (CUGH), Global Health Education Consortium (GHEC), Working Group on Ethics Guidelines for Global Health Training (WEIGHT), and World Health Organization (WHO). Widespread adoption by medical institutions of any single guideline has been limited, however. Moving forward, it will be critical to establish a universally adopted best practice guideline in global health education to assist medical institutions and educators during the development of pre-, intra-, and post-elective training programs.

Our study had several limitations. We acknowledge our small sample size of ten articles. In addition, selection bias and publication bias may have influenced our results. Since individuals typically apply to participate in a global health elective, the data reported here among participants likely represents a unique, self-selected cohort of graduate medical residents who may have been more likely to emphasize positive effects of these experiences. Furthermore, the majority of data reported was qualitative in nature and described self-reported effects.

## Conclusion

Global health electives during residency provide a unique opportunity for visiting trainees to enhance their training and shape their career paths. Our review confirms existing observations that global health electives produce numerous benefits for visiting trainees. We do highlight challenges of participating on abroad electives, specifically health and safety concerns, however. Global health educators should recognize the potential challenges encountered during a global health elective and obtain feedback from their visiting trainees and partner institutions on how to mitigate these concerns. Incorporation of mandatory pre-, intra-, and post-elective training programs and establishment of a universally adopted global health best practice guideline may address some of the challenges visiting trainees encounter while abroad. US medical institutions have a responsibility to develop and implement training programs that will prepare and support visiting trainees to maximize the benefits and minimize the risks of their global health electives.

## References

[1] Koplan JP, Bond TC, Merson MH, et al. Towards a common definition of global health. Lancet. 2009; 373: 1993–5. DOI: 10.1016/S0140-6736(09)60332-9 19493564 PMC9905260

[2] Butteris SM, Schubert CJ, Batra M, et al. Global health education in US pediatric residency programs. Pediatrics. 2015; 136: 458–65. DOI: 10.1542/peds.2015-0792 26260713

[3] Drain PK, Holmes KK, Skeff KM, Hall TL and Gardner P. Global health training and international clinical rotations during residency: current status, needs, and opportunities. Acad Med. 2009; 84: 320–5. DOI: 10.1097/ACM.0b013e3181970a37 19240438 PMC3998377

[4] Torjesen K, Mandalakas A, Kahn R and Duncan B. International child health electives for pediatric residents. Arch Pediatr Adolesc Med. 1999; 153: 1297–302. DOI: 10.1001/archpedi.153.12.1297 10591310

[5] Kolars JC, Halvorsen AJ and McDonald FS. Internal medicine residency directors perspectives on global health experiences. Am J Med. 2011; 124: 881–5. DOI: 10.1016/j.amjmed.2011.04.003 21658664

[6] Dowell J and Merrylees N. Electives: Isn’t it time for a change? Med Educ. 2009; 43: 121–6. DOI: 10.1111/j.1365-2923.2008.03253.x 19161481

[7] Elit L, Hunt M, Redwood-Campbell L, Ranford J, Adelson N and Schwartz L. Ethical issues encountered by medical students during international health electives. Med Educ. 2011; 45: 704–11. DOI: 10.1111/j.1365-2923.2011.03936.x 21649703

[8] Rahim A, Knights Nee Jones F, Fyfe M, Alagarajah J and Baraitser P. Preparing students for the ethical challenges on international health electives: A systematic review of the literature on educational interventions. Med Teach. 2016; 38: 911–20. DOI: 10.3109/0142159X.2015.1132832 26841123

[9] Merlin JS, Morrison G, Gluckman S, et al. Blood and body fluid exposures among US medical students in Botswana. J Gen Intern Med. 2011; 26: 561–4. DOI: 10.1007/s11606-010-1586-4 21116867 PMC3077487

[10] Melby MK, Loh LC, Evert J, Prater C, Lin H and Khan OA. Beyond medical “missions” to impact-drive short-term experiences in global health (STEGHs): Ethical principles to optimize community benefit and learner experience. Acad Med. 2016; 91: 633–8. DOI: 10.1097/ACM.0000000000001009 26630608

[11] Bozinoff N, Corman KP, Kerr D, et al. Toward reciprocity: Host supervisor perspectives on international medical electives. Med Educ. 2014; 48: 397–404. DOI: 10.1111/medu.12386 24606623

[12] Kraeker C and Chandler C. “We learn from them, they learn from us”: Global health experiences and host perceptions of visiting health care professionals. Acad Med. 2013; 88: 483–7. DOI: 10.1097/ACM.0b013e3182857b8a 23425985

[13] Kumwenda B, Dowell J, Daniels K and Merrylees N. Medical electives in sub-Saharan Africa: A host perspective. Med Educ. 2015; 49: 623–33. DOI: 10.1111/medu.12727 25989410

[14] Kung TH, Richardson ET, Mabud TS, Heaney CA, Jones E and Evert J. Host community perspectives on trainees participating in short-term experiences in global health. Med Educ. 2016; 50: 1122–30. DOI: 10.1111/medu.13106 27762010

[15] Lukolyo H, Rees CA, Keating EM, et al. Perceptions and expectations of host country preceptors of short-term learners at four clinical sites in Sub-Saharan Africa. Acad Pediatr. 2016; 16: 387–93. DOI: 10.1016/j.acap.2015.11.002 26581780

[16] O’Donnell S, Adler DH, Inboriboon PC, Alvarado H, Acosta R and Godoy-Monzon D. Perspectives of South American physicians hosting foreign rotators in emergency medicine. Int J Emerg Med. 2014; 7: 24 DOI: 10.1186/s12245-014-0024-5 25635188 PMC4306044

[17] Parekh N, Sawatsky AP, Mbata I, Muula AS and Bui T. Malawian impressions of expatriate physicians: A qualitative study. Malawi Med J. 2016; 28: 43–7. DOI: 10.4314/mmj.v28i2.3 27895827 PMC5117098

[18] Crump JA and Sugarman J. Working Group on Ethics Guidelines for Global Health Training (WEIGHT). Ethics and best practice guidelines for training experiences in global health. Am J Trop Med Hyg. 2010; 83: 1178–82. DOI: 10.4269/ajtmh.2010.10-0527 21118918 PMC2990028

[19] Jogerst K, Callender B, Adams V, et al. Identifying interprofessional global health competences for 21st-century health professionals. Ann Glob Health. 2015; 81: 239–47. DOI: 10.1016/j.aogh.2015.03.006 26088089

[20] Drain PK, Primack A, Hunt DD, Fawzi WW, Holmes KK and Gardner P. Global health in medical education: A call for more training and opportunities. Acad Med. 2007; 82: 226–30. DOI: 10.1097/ACM.0b013e3180305cf9 17327707

[21] Jeffrey J, Dumont RA, Kim GY and Kuo T. Effects of international health electives on medical student learning and career choice: results of a systematic literature review. Fam Med. 2011; 43: 21–8. 21213133

[22] Provenzano AM, Graber LK, Elansary M, Khoshnood K, Rastegar A and Barry M. Short-term global health research projects by US medical students: Ethical challenges for partnerships. Am J Trop Med Hyg. 2010; 83: 211–4. DOI: 10.4269/ajtmh.2010.09-0692 20682858 PMC2911161

[23] Ramsey AH, Haq C, Gjerde CL and Rothenberg D. Career influence of an international health experience during medical school. Fam Med. 2004; 36: 412–6. 15181553

[24] Thompson MJ, Huntington MK, Hunt DD, Pinsky LE and Brodie JJ. Educational effects of international health electives on US and Canadian medical students and residents: A literature review. Acad Med. 2003; 78: 342–7. DOI: 10.1097/00001888-200303000-00023 12634222

[25] Moher D, Liberati A, Tetzlaff J, Altman DG and PRISMA Group. Preferred reporting items for systematic reviews and meta-analysis: The PRISMA statement. PLoS Med. 2009; 6: e1000097 DOI: 10.1371/journal.pmed.1000097 19621072 PMC2707599

[26] Arora G, Perkins KL and Hoffman R. Optimizing global health electives through partnerships: A pilot study of pediatric residents. Acad Pediatr. 2015; 15: 565–7. DOI: 10.1016/j.acap.2015.06.006 26233833

[27] Castillo J, Goldenhar LM, Baker RC, Kahn RS and DeWitt TG. Reflective practice and competencies in global health training: Lesson for servicing diverse patient populations. J Grad Med Educ. 2010; 2: 449–55. DOI: 10.4300/JGME-D-10-00081.1 21976097 PMC2951788

[28] Federico SG, Zachar PA, Oravec CM, Mandler T, Goldson E and Brown J. A successful international child health elective: The University of Colorado Department of Pediatrics’ experience. Arch Pediatr Adolesc Med. 2006; 160: 191–6. DOI: 10.1001/archpedi.160.2.191 16461877

[29] Hau DK, Dipace JI, Peck RN and Johnson WD. Global health training during residency: The Weill Cornell Tanzania experience. J Grad Med Educ. 2011; 3: 421–4. DOI: 10.4300/JGME-D-10-00204.1 22942978 PMC3179217

[30] Nuckton TJ, Luther KA, Weinberger MA, et al. Residency training in developing nations: An international elective for US physicians in training. Teach Learn Med. 1999; 11: 207–13. DOI: 10.1207/S15328015TLM110405

[31] Shull H, Tymchuk C, Grogan T, Hamilton J, Friedman J and Hoffman RM. Evaluation of the UCLA department of medicine Malawi global health clinical elective: Lessons from the first five years. Am J Trop Med Hyg. 2014; 91: 876–80. DOI: 10.4269/ajtmh.14-0147 25223939 PMC4228879

[32] Balmer DF, Marton S, Gillespie SL, Schutze GE and Gill A. Reentry to pediatric residency after global health experiences. Pediatrics. 2015; 136: 680–6. DOI: 10.1542/peds.2015-1255 26391947

[33] Gupta AR, Wells CK, Horwitz RI, Bia FJ and Barry M. The international health program: The fifteen-year experience with the Yale University’s internal medicine residency program. Am J Trop Med Hyg. 1999; 61: 1019–23. DOI: 10.4269/ajtmh.1999.61.1019 10674689

[34] Miller WC, Corey GR, Lallinger GJ and Durack DT. International health and internal medicine residency training: the Duke University experience. Am J Med. 1995; 99: 291–7. DOI: 10.1016/S0002-9343(99)80162-4 7653490

[35] Gladding S, Zink T, Howard C, Campagna A, Slusher T and John C. International electives at the University of Minnesota global pediatric residency program: Opportunities for education in all Accreditation Council for Graduate Medical Education competencies. Acad Pediatr. 2012; 12: 245–50. DOI: 10.1016/j.acap.2012.02.009 22483843

[36] Grudzen CR and Legome E. Loss of international medical experiences: Knowledge, attitudes and skills at risk. BMC Med Educ. 2007; 7: 47 DOI: 10.1186/1472-6920-7-47 18045481 PMC2242732

[37] Sawatsky AP, Rosenman DJ, Merry SP and McDonald FS. Eight years of the Mayo international health program: What an international elective adds to resident education. Mayo Clin Proc. 2010; 85: 734–41. DOI: 10.4065/mcp.2010.0107 20675512 PMC2912735

[38] Bateman C, Baker T, Hoornenborg E and Ericsson U. Bringing global issues to medical teaching. Lancet. 2001; 358: 1539–42. DOI: 10.1016/S0140-6736(01)06586-2 11705584

[39] Brewer TF, Saba N and Clair V. From boutique to basic: A call for standardised medical education in global health. Med Educ. 2009; 43: 930–3. DOI: 10.1111/j.1365-2923.2009.03458.x 19769640

[40] Powell AC, Casey K, Liewehr DJ, Hayanga A, James TA and Cherr GS. Results of a national survey of surgical resident interest in international experience, electives, and volunteerism. J Am Coll Surg. 2009; 208: 304–12. DOI: 10.1016/j.jamcollsurg.2008.10.025 19228545

[41] Smith JK and Weaver DB. Capturing medical students’ idealism. Ann Fam Med. 2006; 4: S32–7. DOI: 10.1370/afm.543 17003160 PMC1578670

[42] Umoren RA, Gardner A, Stone GS, et al. Career choices and global health engagement: 24-year follow-up of US participants in the Indiana University-Moi University elective. Healthc (Amst). 2015; 3: 185–9. DOI: 10.1016/j.hjdsi.2015.10.001 26699341

[43] Reid MJ, Biller N, Lyon SM, et al. Reducing risk and enhancing education: US medical students on global health electives. Am J Infect Control. 2014; 42: 1319–21. DOI: 10.1016/j.ajic.2014.09.007 25465263

[44] Dell EM, Varpio L, Petrosoniak A, Gajaria A and McMcarthy AE. The ethics and safety of medical student global health electives. Int J Med Educ. 2014; 5: 63–72. DOI: 10.5116/ijme.5334.8051 25341214 PMC4207171

[45] Galvin S, Robertson R and Hargarten S. Injuries occurring in medical students during international medical rotations: a strategy toward maximizing safety. Fam Med. 2012; 44: 404–7. 22733417

[46] Hansoti B, Douglass K, Tupesis J, et al. Guidelines for safety of trainees rotating abroad: Consensus recommendations from the Global Emergency Medicine Academy of the Society for Academic Emergency Medicine, Council of Emergency Medicine Residency Directors, and the Emergency Medicine Residents’ Association. Acad Emerg Med. 2013; 20: 413–20. DOI: 10.1111/acem.12106 23701352

[47] Herbst de Cortina S, Arora G, Wells T and Hoffman RM. Evaluation of a structured predeparture orientation at the David Geffen School of Medicine’s global health education programs. Am J Trop Med Hyg. 2016; 94: 563–7. DOI: 10.4269/ajtmh.15-0553 26755562 PMC4775891

[48] Imperato PJ, Bruno DM and Sweeney MM. Ensuring the health, safety, and preparedness of US medical students participating in global health electives overseas. J Community Health. 2016; 41: 442–50. DOI: 10.1007/s10900-016-0169-7 26882901

[49] Purkey E and Hollaar G. Developing consensus for postgraduate global health electives: Definitions, pre-departure training and post-return debriefing. BMC Med Educ. 2016; 16: 159 DOI: 10.1186/s12909-016-0675-4 27259965 PMC4893221

[50] Adams LV and Sosin AN. Beyond visas and vaccines: Preparing students for domestic and global health engagement. Ann Glob Health. 2016; 82: 1056–63. DOI: 10.1016/j.aogh.2016.10.010 28314493

[51] Chase JA and Evert J. Global Health Training in Graduate Medical Education: A Guidebook 2nd ed San Francisco, CA iUniverse; 2011.

[52] Arthur MA, Battat R and Brewer TF. Teaching the basics: Core competencies in global health. Infect Dis Clin North Am. 2011; 25: 347–58. DOI: 10.1016/j.idc.2011.02.013 21628050 PMC7135705

[53] Ablah E, Biberman DA, Weist EM, et al. Improving global health education: Development of a global health competency model. Am J Trop Med Hyg. 2014; 90: 560–5. DOI: 10.4269/ajtmh.13-0537 24445206 PMC3945704

[54] WHO. WHO Global Competency Model. World Health Organization Web site. http://www.who.int/employment/competencies/WHO_competencies_EN.pdf. Accessed January 1, 2017.

